# Molecular and functional characterization of cold-responsive C-repeat binding factors from *Brachypodium distachyon*

**DOI:** 10.1186/1471-2229-14-15

**Published:** 2014-01-09

**Authors:** Jae Yong Ryu, Shin-Young Hong, Sin-Hye Jo, Je-Chang Woo, Sangmin Lee, Chung-Mo Park

**Affiliations:** 1 Department of Chemistry, Seoul National University, Seoul 151-742, Korea; 2 Department of Biological Science, Mokpo National University, Jeonnam 534-729, Korea; 3 Plant Genomics and Breeding Institute, Seoul National University, Seoul 151-742, Korea

**Keywords:** *Brachypodium distachyon*, C-repeat binding factor (CBF), COLD-REGULATED (COR), Abiotic stress tolerance, *Arabidopsis thaliana*

## Abstract

**Background:**

Adverse environmental conditions severely influence various aspects of plant growth and developmental processes, causing worldwide reduction of crop yields. The C-repeat binding factors (CBFs) are critical transcription factors constituting the gene regulatory network that mediates the acclimation process to low temperatures. They regulate a large number of cold-responsive genes, including *COLD-REGULATED* (*COR*) genes, via the CBF-COR regulon. Recent studies have shown that the CBF transcription factors also play a role in plant responses to drought and salt stresses. Putative *CBF* gene homologues and their downstream genes are also present in the genome of *Brachypodium distachyon*, which is perceived as a monocot model in recent years. However, they have not been functionally characterized at the molecular level.

**Results:**

Three *CBF* genes that are responsive to cold were identified from *Brachypodium*, designated *BdCBF1*, *BdCBF2*, and *BdCBF3*, and they were functionally characterized by molecular biological and transgenic approaches in *Brachypodium* and *Arabidopsis thaliana*. Our results demonstrate that the *BdCBF* genes contribute to the tolerance response of *Brachypodium* to cold, drought, and salt stresses by regulating downstream targets, such as *DEHYDRIN5.1* (*Dhn5.1*) and *COR* genes. The *BdCBF* genes are induced under the environmental stress conditions. The BdCBF proteins possess transcriptional activation activity and bind directly to the promoters of the target genes. Transgenic *Brachypodium* plants overexpressing the *BdCBF* genes exhibited enhanced resistance to drought and salt stresses as well as low temperatures, and accordingly endogenous contents of proline and soluble sugars were significantly elevated in the transgenic plants. The BdCBF transcription factors are also functional in the heterologous system *Arabidopsis*. Transgenic *Arabidopsis* plants overexpressing the *BdCBF* genes were also tolerant to freezing, drought, and salt stresses, and a set of stress-responsive genes was upregulated in the transgenic *Arabidopsis* plants.

**Conclusions:**

Taken together, our results strongly support that the BdCBF transcription factors are key regulators of cold stress responses in *Brachypodium* and the CBF-mediated cold stress signaling pathway is conserved in this plant species. We believe that this study would confer great impact on stress biology in monocot species and could be applied to engineer abiotic stress tolerance of bioenergy grass species.

## Background

Plants frequently encounter abrupt environmental changes, such as temperature extremes and drought and salt stresses, in their natural habitats. Therefore, they have developed versatile defense mechanisms to cope with adverse growth conditions, which would underlie the astonishing diversity in plant architecture and physiology [1]. Low temperatures severely influence diverse aspects of plant growth and development, resulting in global loss of crop yields and productivity [2,3]. Upon exposure to low temperatures, plants execute a series of adjustments in molecular genetic, physiological, and behavioral processes, which are termed cold acclimation [4].

During the last decade, numerous genes have been identified and functionally characterized in plant response to low temperatures, and underlying molecular mechanisms and signaling schemes have been established in many plant species, mostly in *Arabidopsis*[5-11]. Cold acclimation enhances plant tolerance to low temperatures by inducing a large set of genes, among which *COLD-REGULATED* (*COR*) genes are best characterized [6]. The promoters of many *COR* genes contain one or more copies of the widely conserved *cis*-acting element, C-repeat/dehydration response element (CRT/DRE) having the CCGAC core sequence [9], indicating that multiple cold responses are coordinately regulated by common regulators.

It has been shown that a small group of transcription factors, designated C-repeat binding factors (CBFs) that belong to the APETALA2/ethylene-response element binding protein (AP2/EREBP) transcription factor family [7,8], bind directly to the promoters of the *COR* genes [8,9]. The *CBF* genes are induced under low temperature conditions. The CBF regulation of *COR* genes constitutes a major cold signaling pathway, termed the CBF-COR regulon [7,9,12,13], which regulates a wide array of cold response genes. The *CBF* gene expression is also modulated by upstream regulators. Recent studies have shown that inducer of CBF expression 1 (ICE1), high expression of osmotically responsive gene 1 (HOS1), and MYB15 function upstream of the *CBF* genes [11,14,15]. ICE1 binds to the *CBF3* gene promoter to induce gene expression [11]. In contrast, MYB15 binds to the *CBF* gene promoters, resulting in suppression of the *CBF* genes [15]. The cold signaling attenuator HOS1 is an E3 ubiquitin ligase that degrades ICE1 and thus compromises extreme cold responses that are harmful to plants [14].

In *Arabidopsis*, six *CBF* genes have been identified, among them *CBF1*, *CBF2,* and *CBF3* are involved in the regulation of cold-related gene expression [7,9]. These three *CBF* genes are induced at low temperatures [8]. They are also induced under other abiotic stress conditions, such as drought and high salt [9,12,13]. In contrast, the *CBF4* gene is not induced at low temperatures but induced under drought and salt stress conditions [10]. Transgenic plants overexpressing *CBF1* or *CBF3* exhibit improved cold tolerance, and *COR* genes are upregulated in the transgenic plants [9,12]. Notably, the transgenic plants are also tolerant to salt and drought stresses [13], suggesting that the CBF function is not restricted to cold acclimation. The *CBF2* gene has been known as a negative regulator of *CBF1* and *CBF3* in *Arabidopsis*[16]. It has been observed that *CBF2*-deficient mutants are tolerant to cold temperatures, and *CBF1* and *CBF3* genes are induced in the mutants [16]. It is likely that the expression of *CBF* genes is elaborately modulated through a negative feedback loop, which may be related with the expression kinetics of the *CBF* genes: gradual reduction of the transcript levels following the peaks right after cold exposure [16].

Recent molecular genetic and bioinformatic studies indicate that the CBF-COR regulon is conserved in a wide variety of monocots and dicots, such as barley, Chinese cabbage, rice, and wheat [17-23], signifying the physiological significance of cold accumulation in plants under fluctuating temperature conditions. *Brachypodium distachyon* is a temperate wild grass species that has been explored as a monocot model for studies on wheat and barley because of their genetic and genomic structural similarities [24]. In addition, its morphological and genetic characteristics, relatively well-established molecular tools, and simple growth requirements make it an ideal model system for bioenergy grass biology [25-28]. A potential target of engineering bioenergy grass, such as switchgrass and *Miscanthus*, is cold stress tolerance, which is intimately linked with grass productivity [29]. However, cold-responsive genes have not been functionally characterized, and related signaling pathways have not been established in *Brachypodium* as well as in bioenergy grass.

In this study, with an aim of extending our understanding on temperature responses in *Brachypodium*, we isolated three potential *CBF* genes (*BdCBF1, BdCBF2,* and *BdCBF3*) that are responsive to cold and functionally characterized their roles in abiotic stress responses via transgenic approaches. Similar to the roles of *CBF* genes in *Arabidopsis*, the *BdCBF* genes was induced under various abiotic stress conditions, and transgenic *Brachypodium* plants were tolerant to cold, drought, and salt stresses. The *BdCBF* genes were also functional in *Arabidopsis*, and cold response genes were upregulated in the transgenic *Arabidopsis* plants that exhibit enhanced tolerance to freezing, drought, and high salt conditions. These observations indicate that the CBF-COR regulon and related cold signaling pathways are conserved in *Brachypodium*.

## Results

### Isolation of *BdCBF* genes

We searched for key regulators of cold responses in the *Brachypodium* genome (http://www.brachypodium.org), with emphasis on CBF homologues. Through BLAST searches using the *Arabidopsis* CBF1, CBF2, and CBF3 proteins as baits, we identified 19 potential CBF homologues that have sequence similarities of higher than 50% throughout the full amino acid sequences to the *Arabidopsis* CBF proteins (Additional file 1). The identified *Brachypodium* CBF homologues have residue numbers ranging from 211 to 295. Recently, it has been shown by microarray assays that many of the *Brachypodium CBF* genes are cold-responsive [30].

Phylogenetic analysis revealed that the CBF homologues are classified into 4 clades (I to IV), among which 4 CBF homologues belonging to clade IV are somewhat distant from those belonging to clades I to III (Additional file 2). In addition, the clade IV members have not been determined whether they are cold-responsive or not [30]. The CBF homologues belonging to clades I to III were designated BdCBFs (Additional files 1 and 2). Clade I contains a single member (Bradi3g51630.1), which was designated BdCBF1. Clade II contains 2 members: Bradi1g49560.1 was designated BdCBF2, and Bradi1g49570.1 as BdCBF2.1. The other 12 BdCBF members belong to clade III, among which Bradi4g35630.1 was designated BdCBF3, and the other members as BdCBF3.1 to BdCBF3.11.

**Figure 1:**
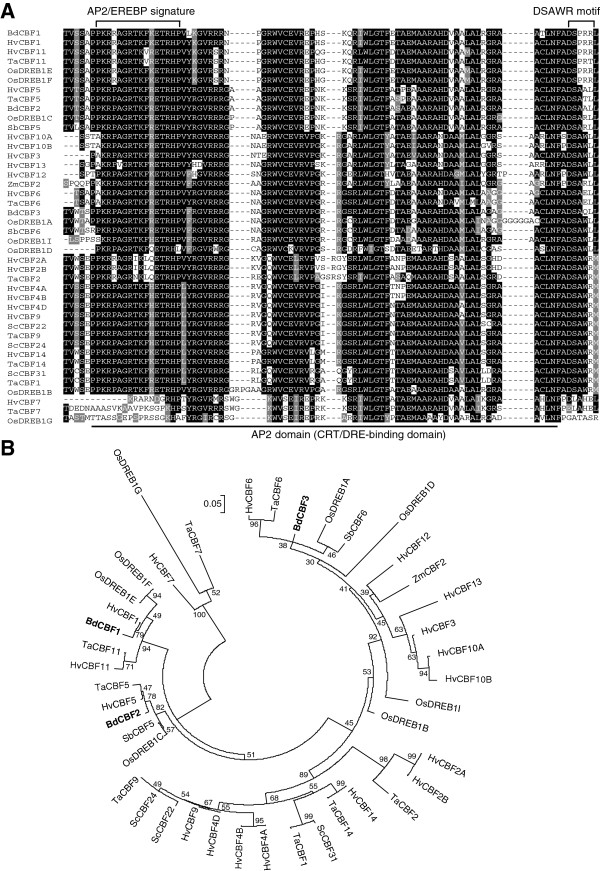
**Amino acid sequence comparison of BdCBFs with monocot CBFs. A**. Multiple sequence alignment of the AP2 domains of CBF proteins. Amino acid sequences of the AP2 domains were aligned using ClustalX (http://www.clustal.org/clustal2/). Black boxes indicate identical residues, and gray boxes indicate biochemically conserved residues. The AP2/EREBP and DSAWR signature sequences are marked by brackets. Abbreviations for plant species are as follows; Bd, *Brachypodium distachyon*; Hv, *Hordeum vulgare*; Os, *Oryza sativa*; Sb, *Sorghum bicolor*; Sc, *Secale cereale*; Ta, *Triticum aestivum*; Zm, *Zea mays*. **B**. Phylogenetic analysis of monocot CBF proteins. The phylogenetic tree was constructed using the MEGA5 software [32]. Bootstrap values (>50%) based on 1000 replicates are shown.

The AP2 DNA-binding domains of the BdCBF1, BdCBF2, and BdCBF3 proteins have sequence identities of higher than 70% with those of monocot CBF proteins (Figure 1A). Phylogenetic analysis revealed that the BdCBF proteins are evolutionally close to those from barley and wheat (Figure 1B), which have been functionally characterized [19,21-23]. The structural conservation and phylogenetic relationship of the CBF proteins would reflect the close genetic relationship between *Brachypodium* and the small grain cereals [31]. Based the sequence analysis data, we hypothesized that the BdCBF proteins, and related cold signaling pathways as well, would be functionally conserved in *Brachypodium*.

### Binding of BdCBF transcription factors to the promoters of cold-responsive genes

The CBF proteins function as transcription factors that bind to the CRT/DRE elements of cold-responsive genes in many plant species [21]. We therefore assumed that the BdCBF proteins possess transcriptional activation activity in the regulation of cold-responsive genes in *Brachypodium*.

**Figure 2:**
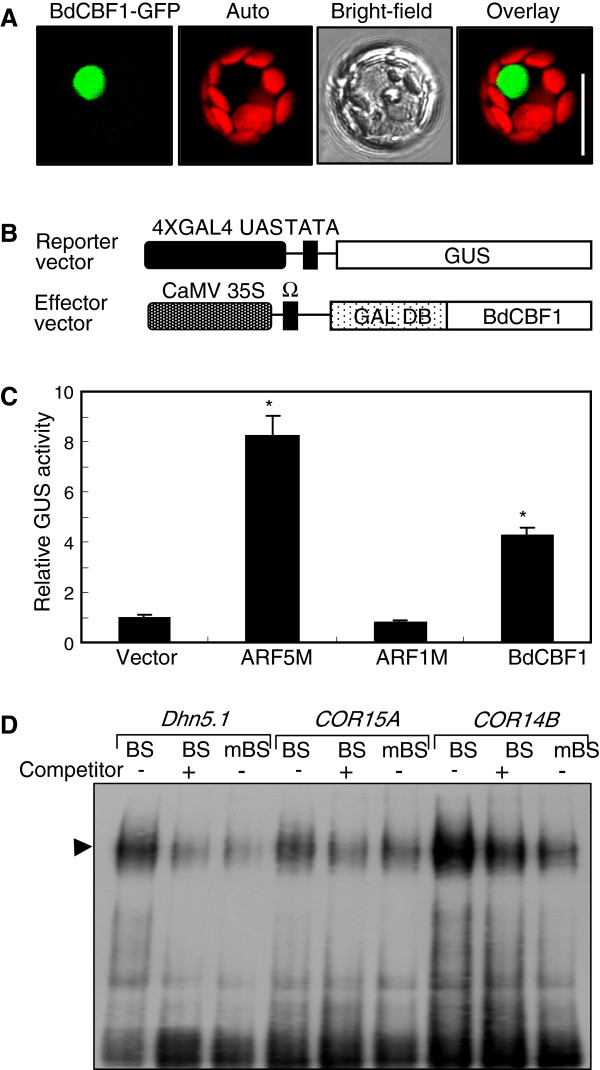
**Transcriptional activation and DNA-binding activities of BdCBF1. A**. Nuclear localization of BdCBF1. The *BdCBF1-GFP* fusion, in which the GFP-coding sequence was fused in-frame to the 3' end of *BdCBF1* cDNA, was expressed transiently in *Brachypodium* protoplasts and visualized by fluorescence microscopy and differential interference contrast microscopy. Auto, autofluorescence. Scale bar = 10 μm. **B** and **C**. Transcriptional activation activity assays in *Brachypodium* protoplasts. The reporter and effector vectors used were diagrammed **(B)**. UAS, upstream sequence; Ω, translational enhancer; DB, DNA binding. The GAL4 transient expression assays were carried out using *Brachypodium* protoplasts **(C)**. Vector control, transformation with the effector vector without gene inserts. ARF5M and ARF1M, transformations with the effector vectors containing *ARF5M* gene (activator control) and *ARF1M* gene (repressor control), respectively [33]. Five measurements were averaged and statistically treated using the Student *t*-test (*P < 0.01). Bars indicate standard error of the mean. **D**. EMSA assays. Recombinant BdCBF1-His fusion protein and radiolabeled DNA were used. Both wild-type and mutated CRT sequences (BS and mBS, respectively) were included in the assays. Excess amounts of unlabeled BS (x100) DNA fragments were also included in the assays to verify specific binding of BdCBF1 to the CRT sequences. Arrowhead indicates protein-DNA complexes. BS, CRT sequence; mBS, mutated BS.

We first examined the subcellular localization of the BdCBF1 protein. A green fluorescence protein (GFP)-coding sequence was fused in-frame to the 3′ end of the *BdCBF1* cDNA sequence, and the *BdCBF1-GFP* gene fusion was expressed transiently in *Brachypodium* protoplasts. The results showed that the BdCBF1 protein is localized exclusively in the nucleus (Figure 2A).

We next examined whether BdCBF1 possesses transcriptional activation activity by employing a GAL4 transient expression system in *Brachypodium* protoplasts [34]. The *BdCBF1* cDNA sequence was fused in-frame to the 3′ end of the GAL4 DNA-binding domain (DB)-coding sequence in the effector vector (Figure 2B). The effector vector, the reporter vector having the β-glucuronidase gene (*GUS*), and the vector containing the *Renilla* luciferase gene, which was included as internal control to normalize the measurements, were cotransformed into *Brachypodium* protoplasts. Measurements of GUS activity showed that BdCBF1 possesses transcriptional activation activity (Figure 2C), indicating that it is a transcriptional activator.

The *Arabidopsis* CBF proteins induce the expression of *COR* genes by directly binding to the CRT motifs within the gene promoters [7,8,17]. We therefore asked whether the BdCBF1 transcription factor binds to the promoter of *COR* genes. Synthetic nucleotides, which cover the CRT motifs in the promoters of the barley *Dhn5.1* and *COR14B* genes and the *Arabidopsis COR15A* gene (Additional file 3), were analyzed in electrophoretic mobility shift assays (EMSA) using recombinant BdCBF1 protein, which was prepared as BdCBF1-His fusion in *Escherichia coli* cells. The EMSA assays revealed that BdCBF1 binds to the CRT sequences (Figure 2D). The BdCBF1 binding to the CRT sequences was reduced in the presence of excess amounts (5X) of unlabeled competitor DNA. In addition, the BdCBF1 binding was significantly reduced when mutated CRT sequences were used. These observations indicate that the BdCBF1 transcription factor binds specifically to the CRT sequences in the *COR* gene promoters.

### Effects of abiotic stresses on *BdCBF* gene expression

Monocot and dicot *CBF* genes are influenced by a variety of abiotic stress conditions, such as cold, drought, and high salinity [4,5,16,18]. We found that the BdCBF1 protein is a *bona fide* transcription factor, which binds to the promoters of cold-responsive genes from *Arabidopsis* and barley, suggesting that the *BdCBF* genes would be affected by abiotic stresses.

**Figure 3:**
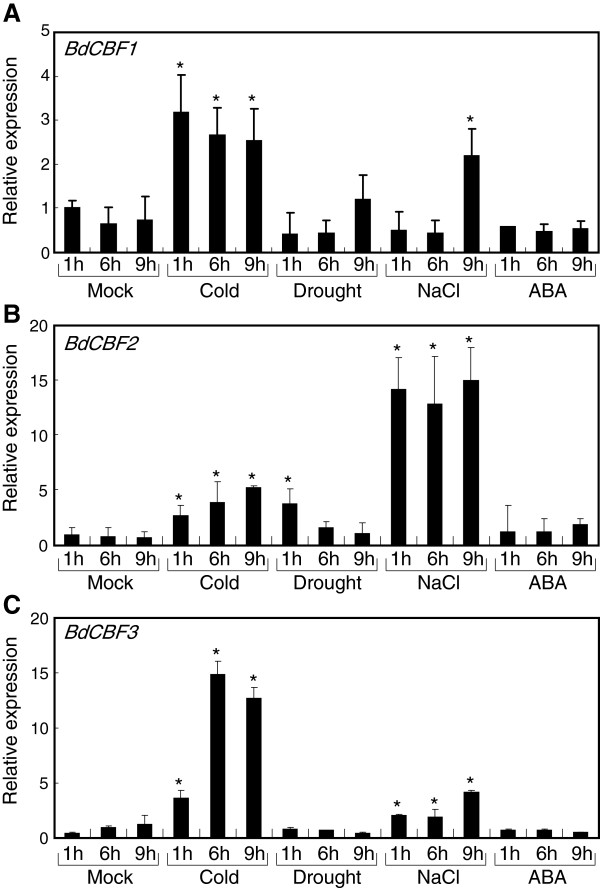
**Effects of abiotic stresses on the expression of *BdCBF *genes.** Two-week-old *Brachypodium* plants grown on MS-agar plates were exposed to cold (4°C) for the indicated time durations. The plants were also floated in MS liquid media containing either 400 mM mannitol, 300 mM NaCl, or 100 μM ABA for the indicated durations. Whole plants were used for the extraction of total RNA samples. Transcript levels were determined by qRT-PCR. Biological triplicates were averaged and statistically treated using the Student *t*-test (*P < 0.01). Bars indicate standard error of the mean. **A**. Effects of abiotic stresses on *BdCBF1* gene expression. **B**. Effects of abiotic stresses on *BdCBF2* gene expression. **C**. Effects of abiotic stresses on *BdCBF3* gene expression.

Two-week-old *Brachypodium* plants were exposed to various abiotic stress conditions, and total RNA was extracted from whole plants for quantitative real-time RT-PCR (qRT-PCR) assays. Gene expression analysis showed that the *BdCBF1*, *BdCBF2*, and *BdCBF3* genes were markedly induced under cold temperature conditions (Figures 3A,B and C). The cold effects were most prominent on the *BdCBF3* gene expression with approximately 15-fold induction. The *BdCBF1* and *BdCBF2* genes were also induced by drought and high salinity. Whereas the *BdCBF3* gene was induced by high salinity, it was not influenced to a detectable level by drought. It is notable that the *BdCBF2* gene is affected most prominently by high salinity among the abiotic stress conditions examined (Figure 3B). Abscisic acid (ABA), which is a major stress hormone in plants [35], did not have any discernible effects on the *BdCBF* gene expression, unlike the *CBF* gene expression patterns in *Arabidopsis* and other plant species [5,21]. These observations suggest that the *BdCBF* genes mediate both cold response and plant responses to drought and salt stress, possibly in an ABA-independent manner.

### Stress-tolerant phenotypes of *BdCBF*-overexpressing *Brachypodium* plants

Based on the expression analysis of *BdCBF* genes under abiotic stress conditions, we postulated that the *BdCBF* genes are associated with plant responses to abiotic stresses.

To investigate the physiological roles of the BdCBF1 transcription factors, the *BdCBF1* cDNA sequence was transformed into *Brachypodium distachyon* ecotype Bd21-3 under the control of the Cauliflower Mosaic Virus (CaMV) 35S promoter. The 35S:*BdCBF1* transgenic *Brachypodium* plants were grown under abiotic stress conditions to examine their stress responsiveness.

**Figure 4:**
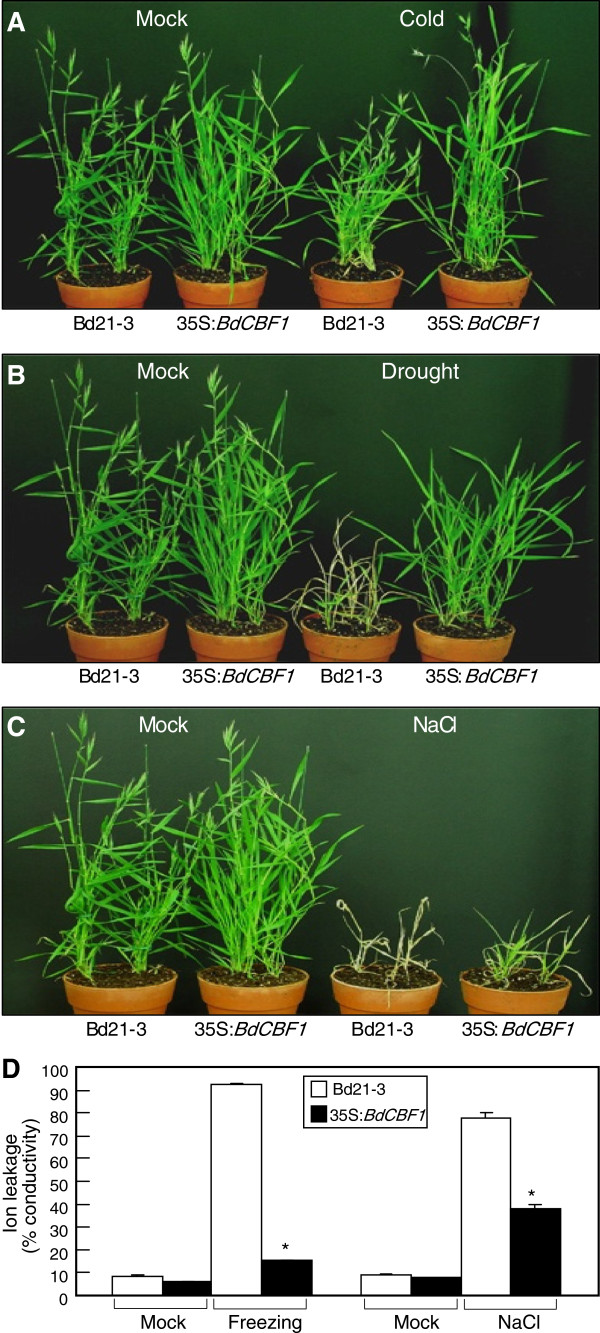
**Stress tolerance of 35S: *BdCBF1 *transgenic *Brachypodium *plants.** The *BdCBF1* cDNA was transformed into *Brachypodium* Bd21-3 ecotype under the control of the CaMV 35S promoter. Six-week-old plants grown in soil were exposed to either cold (4°C for 6 days), drought (for 9 days without watering), or high salinity (300 mM for 3 days) conditions and allowed to recover by growing under normal growth conditions for 15 days before taking photographs. **A**. Cold stress tolerance. **B**. Drought stress tolerance. **C**. Salt stress tolerance. **D**. Electrolyte leakage assays. Two-week-old *Brachypodim* plants were exposed to either freezing (-6°C) for 3 h or high salinity (300 mM NaCl) for 6 h. Rosette leaves were used for the measurements of electrolyte leakage. Five measurements were averaged and statistically treated using the Student *t*-test (*P < 0.01). Bars indicate standard error of the mean.

The 35S:*BdCBF1* transgenic plants were phenotypically indistinguishable from the Bd21-3 control plants except elevated transcription levels of BdCBF1 gene when grown under normal conditions (Figure 4 and Additional file 4). In contrast, they exhibited enhanced tolerance to cold temperatures (4°C), drought, and salt stress (300 mM NaCl) (Figures 4A,B and C, respectively), which is consistent with the inductive effects of the abiotic stresses on the *BdCBF* gene expression. Electrolyte leakage assays using the leaf blades showed that electrical conductivity was drastically reduced in the leaves of the 35S:*BdCBF1* transgenic plants that were treated with freezing temperatures (-6°C/3 h) or high salt (Figure 4D). These results further support the notion that the physiological roles of the BdCBF proteins are similar to those of the CBF proteins in *Arabidopsis* and other plant species.

### Accumulation of proline and sugars in 35S:*BdCBF1* transgenic *Brachypodium* plants

Under abiotic stress conditions, a variety of osmolytes or compatible solutes, such as proline and soluble sugars, accumulates in plant tissues and acts as osmoprotectants that protect plants from extreme osmotic changes [36-39]. In *Arabidopsis*, transgenic plants overexpressing *CBF3* gene are tolerant to freezing temperatures [40]. Proline and sugars accumulate to a high level in the transgenic plants under both normal and cold stress conditions. Since the 35S:*BdCBF1* transgenic *Brachypodium* plants exhibited enhanced tolerance to abiotic stresses, we predicted that osmolytes would accumulate in the transgenic plants.

**Figure 5:**
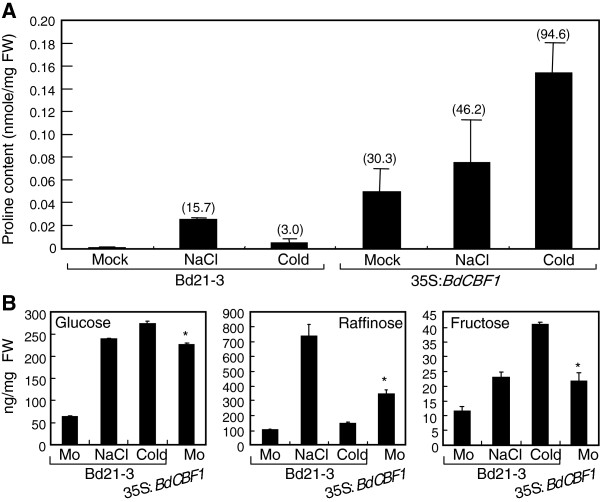
**Contents of proline and sugars in 35S: *BdCBF1 *transgenic *Brachypodium *plants.** Five measurements were averaged and statistically treated using the Student *t*-test (*P < 0.01). Bars indicate standard error of the mean. **A**. Proline contents. Two-week-old plants grown on MS-agar plates were exposed to either 300 mM NaCl for 24 h or 4°C for 24 h. Whole plants were used for the extraction of proline. Contents of free proline were measured by HPLC, as described previously [41]. The numbers in parentheses indicate fold changes relative to the value in the mock-treated Bd21-3 plants. Bars indicate standard error of the mean. **B**. Sugar contents. The plant materials described in **(A)** were used for the extraction of soluble sugars. Contents of individual sugars were determined by HPLC, as described previously [38]. Mo, mock.

Measurements of proline contents showed that the level was higher by 30-fold in the transgenic plants in comparison to that in the Bd21-3 control plants under normal conditions (Figure 5A). The proline levels were further elevated in the transgenic plants when they were exposed to cold and salt stresses, which is in correlation with the enhanced stress tolerance of the transgenic plants. Particularly, under cold stress conditions, whereas the proline level was elevated by 3-fold in control plants, it was elevated by approximately 95-fold in the transgenic plants, sustaining the role of BdCBF1 primarily in cold stress response.

We found that the levels of soluble sugars, such as glucose, raffinose, and fructose, were elevated by 3 ~ 7 times in control plants when exposed to cold and salt stresses (Figure 5B). Notably, the sugar levels were higher in the 35S:*BdCBF1* transgenic plants and comparable to the levels in control plants that were exposed to freezing temperatures and salt stress. It is therefore concluded that the elevated levels of osmolytes at least in part underlie the enhanced tolerance to abiotic stresses in the 35S:*BdCBF1* transgenic *Brachypodium* plants.

### Abiotic stress tolerance in *BdCBF1*-overexpressing *Arabidopsis* plants

To further characterize the functional conservation of the BdCBF transcription factors in different plant species, we produced transgenic *Arabidopsis* plants overexpressing the *BdCBF1* gene under the control of the CaMV35S promoter. Two independent transgenic lines were selected for further analysis according to the high-level expression of the transgene (Additional file 5).

**Figure 6:**
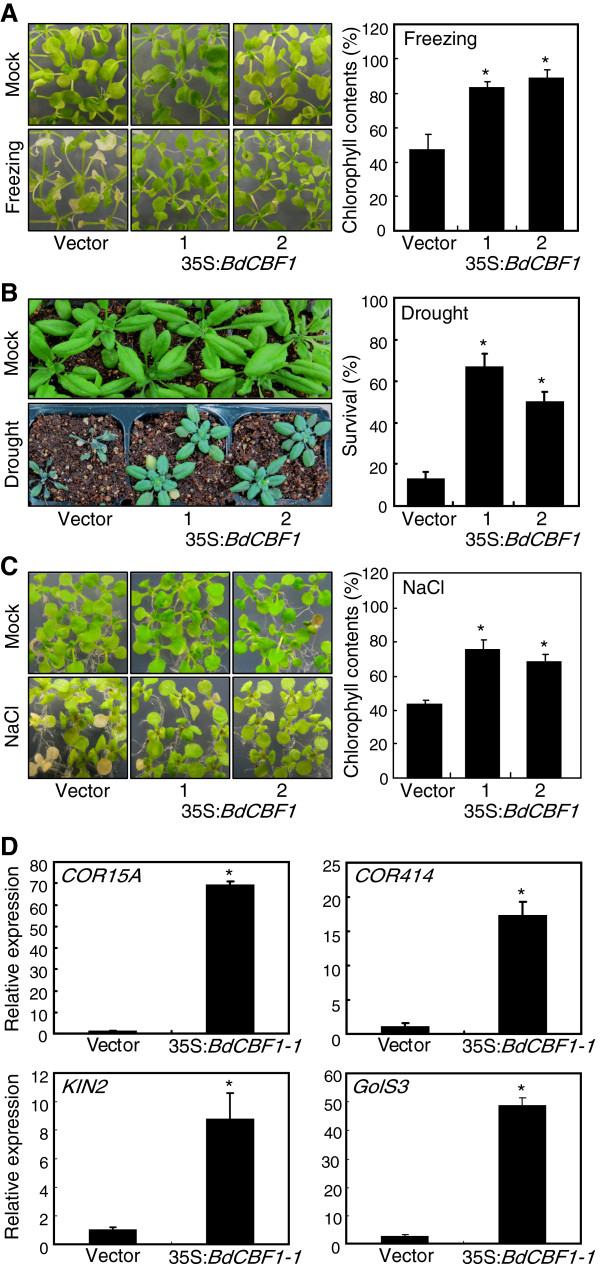
**Stress tolerance of *Arabidopsis *plants overexpressing *BdCBF1 *gene.** The *BdCBF1* cDNA was transformed into *Arabidopsis* Col-0 plants under the control of the CaMV 35S promoter. Two independent transgenic lines were included in the assays. Plants were grown on MS-agar plates for either one **(A and C)** or two weeks **(B)** before stress treatments. In **(A)** to **(C)**, measurements of 30–35 plants were averaged and statistically treated using the Student *t*-test (*P < 0.01). **A**. Freezing tolerance assays. Plants were exposed to -8°C for 5 h and allowed to recover at 23°C for 10 days. Rosette leaves were for the measurements of chlorophyll contents. **B**. Drought tolerance assays. Plants were exposed to drought stress conditions without watering for 12 days and rewatered for 3 additional days before the assays. **C**. Salt tolerance assays. Plants were transferred to MS-agar plates containing 150 mM NaCl and further grown for 5 days. Rosette leaves were for the measurements of chlorophyll contents. **D**. Expression of CBF target genes in 35S:*BdCBF1* transgenic *Arabidopsis* plants. Transcript levels were determined by qRT-PCR. Biological triplicates were averaged and statistically treated using the Student *t*-test (*P < 0.01). Bars indicate standard error of the mean.

The 35S:*BdCBF1* transgenic *Arabidopsis* plants did not show any discernible phenotypic changes when grown under normal conditions (Figure 6). However, they exhibited distinct phenotypes when exposed to stress conditions. For freezing tolerance assays, two-week-old plants grown on ½ X Murashige and Skoog-agar plates (hereafter referred to as MS-agar plates) were exposed to -8°C for 5 h and allowed to recover at 23°C for 10 days. The 35S:*BdCBF1* transgenic plants exhibited enhanced tolerance to freezing temperatures (Figure 6A, left panel). To access the degree of freezing tolerance, chlorophyll contents were measured using the rosette leaves. The levels of chlorophylls were higher by approximately 2-fold in the transgenic plants in comparison to Col-0 plants transformed with vector alone (Figure 6A, right panel).

To investigate drought stress tolerance in the 35S:*BdCBF1* transgenic *Arabidopsis* plants, two-week-old plants grown in soil were further grown without watering for 12 days, when most of the transgenic plants wilted, and rewatered for 3 days before measuring survival rates. The survival rates were significantly higher in the transgenic plants (Figure 6B): whereas the survival rates of the transgenic plants were ~60%, that of control plants was 12% under our assay conditions.

To examine the tolerance response of the transgenic plants under high salinity, one-week-old plants grown on MS-agar plates were transferred to fresh MS-agar plates containing 150 mM NaCl and further grown for 5 days. The 35S:*BdCBF1* transgenic *Arabidopsis* plants were clearly more tolerant to salt stress (Figure 6C, left panel). The chlorophyll levels were approximately 70% higher in the transgenic leaves in comparison to the control leaves (Figures 6C, right panel).

Consistent with the improved tolerance to abiotic stresses in the 35S:*BdCBF1* transgenic *Arabidopsis* plants, the transcript levels of stress response genes, such as *COR15A*, *COR414*, *COR6.6*/*KIN2*, and *Galactinol synthase 3* (*GolS3*), were significantly higher in the transgenic *Arabidopsis* plants (Figure 6D).

Together, our data demonstrate that the *Brachypodium* BdCBF transcription factors are also functional in *Arabidopsis*, further supporting the functional conservation of the CBF-COR cold signaling module in *Brachypodium*.

## Discussion

Many plant species from temperate regions have the ability to acclimate to cold, nonfreezing temperatures, which is essential for plant survival at freezing temperatures. During the cold acclimation process, diverse biochemical and physiological adjustments occur in plant cells. The cold-responsive cellular events include modifications of lipid composition in the cellular membranes and accumulation of osmoprotectants, including anti-freeze proteins, proline, and sugars [42-45].

Cold acclimation is associated with extensive changes in the transcriptome, among which changes in the expression patterns of transcription factor genes are most prevailing [5,6]. In *Arabidopsis*, a central cold signaling pathway includes the CBF transcription factors that directly regulate an array of *COR* genes through the supposed ICE1-CBF-COR regulon [46-48]. Consequently, genes constituting the cold signaling pathway and underlined molecular mechanisms have been comprehensively studied in *Arabidopsis*[2,4-6,11].

The *CBF* genes have been functionally characterized using gene-deficient mutants and transgenic plants overexpressing them in several plant species [7-9,12,16]. Numerous *CBF* gene homologues have been identified through genomic sequence analysis in both monocots and dicots [17,19,49]. Accumulating evidence in recent years indicate that the CBF signaling pathways are conserved in diverse plant species.

The *Brachypodium* genome contains more than a hundred of AP2 domain-containing proteins, among which 14 members have been identified as CBF3 homologues by BLAST searches and phylogenetic analyses [30]. Through similar approaches, we identified 15 putative BdCBF proteins that are classified into 3 clades. Clades I and II contain one and two BdCBF members, respectively. Clade III contains 12 BdCBF members, similar to what have been identified previously [30]. Based on the phylogenetic analyses, we selected one member that is cold responsive from each clade, and the selected BdCBF proteins (BdCBF1-3) were functionally studied in cold response and other abiotic stress responses using transgenic *Brachypodium* and *Arabidopsis* plants.

The predicted amino acid sequences of the BdCBF1 proteins are highly homologous to those in other plant species, particularly to those in wheat, barley, and rice. The AP2 DNA-binding domains of the BdCBF proteins have the highest sequence identity to those in these plant species among others. In *Arabidopsis*, CBF1, CBF2 and CBF3 bind to the CRT/DRE elements of *COR* genes. We found that the BdCBF1 protein binds specifically to the CRT motif within the *COR* gene promoters from barley and *Arabidopsis*, sustaining the functional conservation of BdCBF1, and perhaps BdCBF2 and BdCBF3 as well, in *Brachypodium*.

Expression patterns of the *BdCBF* genes under abiotic stress conditions were quite similar to what observed in *Arabidopsis*[7,8]. The *BdCBF1*, *BdCBF2* and *BdCBF3* genes were induced by cold, high salinity, and drought but with some degree of variations. One distinction was that the *BdCBF* genes were not influenced by ABA. It has been reported that ABA induces the transcription of *CBF* genes, leading to subsequent induction of *COR* genes in *Arabidopsis*[50]. Although further studies are required to confirm the relationship between ABA signaling and *BdCBF* gene expression, molecular mechanisms underlying the stress induction of the *BdCBF* genes would be somewhat different in different plant species. This distinction may be related with recent findings, in which it has been observed that the CBF proteins have both common and distinct roles in different plant species [17-23].

The correlation between proline accumulation and induction of low temperature tolerance has been demonstrated in maize [51]. The roles of proline and soluble sugars as osmoprotectants in low temperature tolerance have also been demonstrated in *Arabidopsis* and rice plants overexpressing *CBF3* gene [40]. The *GolS3* gene, which encodes galactinol synthase catalyzing the first step of the biosynthesis of raffinose family oligosaccharides [52], was induced in the *CBF3*-overexpressing transgenic *Arabidopsis* plants [39]. We found that the levels of proline and soluble sugars were significantly elevated in the 35S:*BdCBF* transgenic *Brachypodium* plants that exhibit enhanced stress tolerance. Abiotic stress tolerance was also improved in the transgenic *Arabidopsis* plants overexpressing the *BdCBF* genes, and *COR* genes, such as *COR15A*, *COR414*, *KIN2*, and *GolS3* that are directly regulated by CBF transcription factors, were markedly upregulated in the transgenic *Arabidopsis* plants [53]. Together with the sequence similarities and phylogenetic relationships of the BdCBF1, BdCBF2, and BdCBF3 proteins to the CBF proteins in *Arabidopsis* and monocot plants, our data on the functional assays strongly support that the CBF proteins and associated CBF-mediated signaling pathways are conserved in *Brachypodium*.

Improvement of stress tolerance is a potential target of genetic engineering in bioenergy grass, such as switchgrass and *Miscanthus*[29,54]. Based on the genetic similarity between the grass species and *Brachypodium*, it is envisioned that the BdCBF-COR regulon would readily be applicable to the bioenergy grass species as efficient transformation systems are to be established in near future.

## Conclusions

We identified three *CBF* gene homologues (*BdCBF*1, *BdCBF2*, and *BdCBF3*) encoding potential transcription factors in the *Brachypodium distachyon* genome and functionally characterized the BdCBF transcription factors using *BdCBF*-overexpressing transgenic *Brachypodium* and *Arabidopsis* plants that exhibit enhanced tolerance to cold temperatures and drought and salt stresses. Proline and soluble sugars accumulated to high levels in the transgenic *Brachypodium* plants. A set of *COR* genes were upregulated in the transgenic *Arabidopsis* plants. It is therefore concluded that the CBF-mediated cold signaling pathway is conserved in *Brachypodium*.

## Methods

### Plant materials and growth conditions

*Brachypodium distachyon* ecotype Bd21-3, which is a community standard diploid inbred line, was used in all experiments. *Brachypodium* plants were grown in a controlled growth chamber with relative humidity of 60% under long day conditions (20-h light/4-h dark). Growth conditions were 24°C during the day and 18°C at night with white light illumination provided by FLR40D/A fluorescent tubes (150 μmol photons/m^2^s, Osram, Seoul, Korea).

The *Arabidopsis* ecotype Columbia (Col-0) was used for *BdCBF* gene transformation. *Arabidopsis* plants were grown in a controlled culture room at 22°C with relative humidity of 55% under long day conditions (16-h light/8-h dark) with white light illumination provided by fluorescent FLR40D/A tubes (120 μmol photons/m^2^s, Osram).

### Plant transformation

To produce transgenic *Brachypodium* and *Arabidopsis* plants overexpressing *BdCBF1* cDNA, the *BdCBF1* cDNA was amplified from a *Brachypodium* cDNA pool and subcloned into the binary pJJ461 vector under the control of the CaMV 35S promoter. *Brachypodium* transformation was performed according to the *Agrobacterium tumefaciens*-mediated method using compact embryogenic calli derived from immature embryos [55]. *A. tumefaciens*-mediated *Arabidopsis* transformation was performed according to a modified floral dip method [56].

### Analysis of gene transcript levels

Total RNA was extracted from plant materials using the RNeasy Plant Mini Kit (Qiagen, Valencia, CA) according to the manufacturer’s procedure. Before RT-PCR and qRT-PCR, total RNA samples were pretreated with a RNase-free DNase I to eliminate contaminating genomic DNA. Primary cDNA was synthesized from approximately 2 μg of total RNA using the MMLV first-strand synthesis system (Promega, Madison, WI).

One μl of the primary cDNA synthesis reaction mixture (20 μl) was taken for subsequent PCR amplification. RT-PCR runs consisted of 15–35 cycles, depending on the linear range of PCR amplification for individual genes. Each PCR cycle included incubations at 94°C for 0.5 min, at 55°C for 0.5 min and at 72°C for 3 min. One additional cycle at 72°C for 7 min was included after the last cycle to allow completion of incomplete polymerizations.

qRT-PCR was performed in 96-well blocks with the Applied Biosystems 7500 Real-Time PCR System (Foster City, CA) using the SYBR Green I master mix in a volume of 20 μl. The PCR primers were designed using the Primer Express software installed in the system and listed in (Additional file 6). The two-step thermal cycling profile and processing of qRT-PCR data were performed as described previously [57]. For the accurate measurements of gene transcript levels, biological triplicates were averaged and statistically treated using the Student *t*-test.

### BLAST searches and phylogenetic analyses

To identify putative CBF homologues in the *Brachypodium* genome, the BLAST search tool was employed (http://blast.ncbi.nlm.nih.gov/Blast.cgi). BLAST searches were performed using the *Arabidopsis* CBF1, CBF2, and CBF3 proteins as baits. Nineteen CBF homologues having higher than 50% amino acid sequence similarities throughout the full sequences were selected for further studies. Phylogenetic analyses were performed on the 19 *Brachypodium* CBF homologues using the MEGA5 software, as described previously [32], and 15 members of them were designated BdCBF proteins.

To investigate the phylogenetic relationship between the BdCBF1, BdCBF2, and BdCBF3 proteins and those from other monocots, amino acid sequences of putative CBF proteins of *Hordeum vulgare*, *Oryza sativa*, *Sorghum bicolor*, *Secale cereale*, *Triticum aestivum*, and *Zea mays* were obtained from the Plant Genome Database (PlantGDB, http://www.plantgdb.org/) and National Center for Biotechnology Information (NCBI, http://www.ncbi.nlm.nih.gov/).

### Abiotic stress treatments

To examine the effects of abiotic stresses on *BdCBF* gene expression in *Brachypodium*, two-week-old plants grown on MS-agar plates were transferred to MS liquid cultures supplemented with either 400 mM mannitol, 300 mM NaCl, or 100 μM ABA, and incubated for various time durations with gentle shaking. For cold treatments, plants were exposed to 4°C for various time durations before harvesting plant materials.

To examine the effects of abiotic stresses on *Brachypodium* growth, six-week-old plants grown in soil were used. For cold treatments, plants were exposed to 4°C for 6 days and transferred to normal growth conditions for 15 days. For drought treatments, plants were grown for 9 days without watering, when symptoms of wilting are evident in most of 35S:*BdCBF1* transgenic *Brachypodium* plants, and transferred to normal growth conditions with rewatering for 15 days. For salt stress treatments, plants were watered with 300 mM NaCl solution for 3 days, and the soil was extensively washed with deionized water. The salt-treated plants were further grown under normal growth conditions for 15 days.

For the assays on freezing tolerance in *Arabidopsis*, one-week-old plants grown on MS-agar plates were exposed to -8°C for 5 h and allowed to recover at 23°C for 10 days. For the assays on drought tolerance in *Arabidopsis*, two-week-old plants grown in soil were subsequently grown for 12 days without watering until symptoms of wilting are visible in most of 35S:*BdCBF1* transgenic *Arabidopsis* plants. Survival rates were calculated 3 days after rewatering. For the assays on salt tolerance in *Arabidopsis*, one-week-old plants grown on MS-agar plates were transferred to MS-agar plates supplemented with 150 mM NaCl and further grown for 5 days.

The survival rates were calculated by counting 30–35 plants for each plant genotype and statistically treated using the Student *t*-test.

### Subcellular localization of BdCBF1

The GFP-coding sequence was fused in-frame to the 3′ end of *BdCBF1* cDNA sequence, and the gene fusion was expressed transiently in *Brachypodium* protoplasts, as described previously [34]. After incubation for 16 h at room temperature in complete darkness, the protoplasts were observed using the Multi-photon Confocal Laser Scanning microscope (Carl Zeiss, Jena, Germany).

### Transcriptional activation activity assays

Transient expression assays in *Brachypodium* protoplasts were employed to examine the transcriptional activation activity of BdCBF1. The reporter and effector plasmids were constructed as follows. The reporter plasmid contained four copies of the GAL4 upstream activation sequence and the *GUS* reporter gene. To construct the p35S:BdCBF1 effector plasmid, *BdCBF1* cDNA was fused to the GAL4 DB-coding sequence and inserted into an expression vector containing the CaMV 35S promoter. The reporter and effector plasmids were cotransformed into *Brachypodium* protoplasts by a polyethylene glycol-mediated transformation method [34]. GUS activity was measured by the fluorometric method, as described previously [34]. A CaMV 35S promoter-luciferase construct was also cotransformed as internal control. The luciferase assay was performed using the Luciferase Assay System (Promega). Five measurements were averaged and statistically treated using the Student *t*-test (*P < 0.01) for each assay.

### EMSA assays

The *BdCBF1* cDNA was subcloned into the pET-28a *E. coli* expression vector having a His-coding sequence (Novagen, Darmstadt, Germany). The *BdCBF1-His* fusion was expressed in BL21(DE3)pLysS *E. coli* cells and purified using Ni-NTA agarose beads (Qiagen, Venlo, Netherlands). DNA fragments were end-labeled with [γ-^32^P]dATP using T4 polynucleotide kinase. Labeled probes were incubated with ~0.5 μg of purified recombinant BdCBF1-His proteins for 30 min at 25°C in binding buffer (10 mM Tris-Cl, pH7.6, 50 mM NaCl, 1 mM EDTA, 5 mM DTT, and 5% glycerol). The reaction mixtures were electrophoresed on 6% native PAGE gels. The gels were dried on Whatman 3MM paper and exposed to X-ray films.

### Measurements of proline and sugar contents

For the measurement of endogenous contents of proline and sugars in *Brachypodium*, two-week-old plants grown on MS-agar plates were exposed to 300 mM NaCl for 24 h or 4°C for 24 h. Whole plants were quick-frozen in liquid nitrogen and homogenized. The homogenate was extracted in 80% methanol and boiled for 10 min. The contents of proline and soluble sugars were quantified by high performance liquid chromatography at the National Instrumentation Center for Environmental Management (NICEM), College of Agriculture and Life Sciences, Seoul National University, Seoul, Korea. Five measurements were averaged and statistically treated using the Student *t*-test for each assay.

### Electrolyte leakage assays

To evaluate the degree of cellular injury by freezing temperatures and high salinity in *Brachypodium*, the percentage of electrolyte leakage was measured. Two-week-old plants grown on MS-agar plates were either exposed to -6°C for 3 h or soaked in MS liquid medium containing 300 mM NaCl for 6 h. The aerial plant parts were briefly washed with deionized water and soaked in deionized water for 12 h in complete darkness before measuring sample conductivity using the Orion 5-star conductivity meter (Thermo, Beverly, MA). The plant materials were then boiled in the same solution for 5 min, and total conductivity of the solution was measured. Electrolyte leakage is represented by the relative conductivity that is calculated by dividing sample conductivity by total conductivity. Five measurements were averaged and statistically treated using the Student *t*-test for each assay.

### Measurements of chlorophyll contents

Measurements of chlorophyll contents in *Arabidopsis* were performed as described previously [58]. Chlorophylls were extracted with N,N-dimethylformamide from the rosette leaves, and the extracted solution was incubated at 4°C for 2 h in complete darkness. Chlorophyll contents were assayed by measuring absorbance at 652 nm, 665 nm, and 750 nm using a diode array spectrophotometer (WPA Biowave, Cambridge, UK). Five measurements were averaged and statistically treated using the Student *t*-test for each assay.

## Competing interests

The authors declare that they have no competing interests.

## Authors’ contributions

CMP and SYH conceptualized the project and analyzed the data. CMP, JYR, and SL wrote the manuscript. SYH, JYR, SHJ, and JCW carried out the molecular and biochemical assays on *Brachypodium distachyon*. SL and JYR transformed the *BdCBF1* gene into *Arabidopsis thaliana* and analyzed the stress response of the transgenic plants. All authors discussed the results and approved the final form of the manuscript.

## Supplementary Material

Additional file 1 **Potential CBF homologues in *Brachypodium distachyon*.** Potential CBF homologues were identified in *Brachypodium distachyon* by BLAST searches using the *Arabidopsis* CBF1, CBF2, and CBF3 proteins as baits. The *Brachypodium* proteins having amino acid sequence similarity of higher than 50% are listed. Gene names were given to 15 members of the 19 CBF homologues according to phylogenetic analysis data (see Additional file 2). *The cold responsiveness of each homologue has been determined by microarray assays [30]. That of the BdCBF1, BdCBF2, BdCBF3 proteins were determined by RT-PCR (this work). ND, not determined. Click here for file

Additional file 2 **Phylogenetic analyses of *Brachypodium *** **CBF homologues.** The phylogenetic relationship of the *Brachypodium* CBF homologues was analyzed using the MEGA5 software [32]. The *Brachypodium* CBF homologues were classified into 4 clades (I, II, III, and IV), among which the clade IV members are somewhat distant from the CBF homologues belonging to clades I to III. We selected one homologue that is cold-responsive from each clade and named BdCBF1, BdCBF2, and BdCBF3, as listed in bold. The remaining BdCBF proteins within each clade were serially numbered, like 2.1, 3.1, 3.2, etc. (also see Additional file 1). Click here for file

Additional file 3 **C-repeat (CRT) elements from the barley *Dhn5.1 *** **and *COR14B *** **promoters and the *Arabidopsis COR15A *** **promoter.** The core CRT sequence (CCGAC), designated BS, was mutated to AAATA (mBS), as underlined, for electrophoretic mobility shift assays. Click here for file

Additional file 4 **Expression of *BdCBF1 *** **gene in 35S: *BdCBF1 *** **transgenic *Brachypodium *** **plant.** Transcript levels of *BdCBF1* gene were determined by quantitative real-time RT-PCR (qRT-PCR) using total RNA samples extracted from 14-day-old whole plants grown on ½ X Murashige and Skoog-agar plates (hereafter referred to as MS-agar plates). Biological triplicates were averaged and statistically treated using the Student *t*-test (*P < 0.01). Bars indicate standard error of the mean. Click here for file

Additional file 5 **Expression of *BdCBF1 *** **gene in 35S: *BdCBF1 *** **transgenic *Arabidopsis *** **plants.** Transcript levels of *BdCBF1* gene were determined by RT-PCR using total RNA samples extracted from 10-day-old whole plants grown on MS-agar Plates. A tubulin gene (*TUB*) was used as RNA quality control. Click here for file

Additional file 6 **Primers used in qRT-PCR and RT-PCR.** F, forward primer; R, reverse primer. Click here for file
