# Supplementary material for: Molecular and functional characterization of cold-responsive C-repeat binding factors from Brachypodium distachyon
Source: BMC Plant Biol. 2014 Jan 9;14:15. doi: 10.1186/1471-2229-14-15 (PMC3898008; doi:10.1186/1471-2229-14-15)
Supplement: Additional file 1 — Potential CBF homologues in Brachypodium distachyon . Potential CBF homologues were identified in Brachypodium distachyon by BLAST searches using the Arabidopsis CBF1, CBF2, and CBF3 proteins as baits. The Brachypodium proteins having amino acid sequence similarity of higher than 50% are listed. Gene names were given to 15 members of the 19 CBF homologues according to phylogenetic analysis data (see Additional file 2). *The cold responsiveness of each homologue has been determined by microarray assays [30]. That of the BdCBF1, BdCBF2, BdCBF3 proteins were determined by RT-PCR (this work). ND, not determined. [file 1471-2229-14-15-S1.pdf]

Additional file 1

Gene ID	Gene name	Residue number	Cold response*	Reference
Bradi3g51630	**BdCBF1**	228	O	This work
Bradi1g49560	**BdCBF2**	221	O	This work
Bradi1g49570	BdCBF2.1	224	ND	
Bradi4g35650	**BdCBF3**	239	O	[Ref 30], This work
Bradi4g35600	BdCBF3.1	246	O	[Ref 30]
Bradi1g57970	BdCBF3.2	242	X	[Ref 30]
Bradi4g35590	BdCBF3.3	248	O	[Ref 30]
Bradi4g35610	BdCBF3.4	244	O	[Ref 30]
Bradi4g35620	BdCBF3.5	253	O	[Ref 30]
Bradi4g35630	BdCBF3.6	254	O	[Ref 30]
Bradi4g35570	BdCBF3.7	244	O	[Ref 30]
Bradi3g57360	BdCBF3.8	236	X	[Ref 30]
Bradi2g60340	BdCBF3.9	241	O	[Ref 30]
Bradi4g35580	BdCBF3.10	258	ND	
Bradi1g77120	BdCBF3.11	256	O	[Ref 30]
Bradi3g50630		237	ND	
Bradi5g17610		295	ND	
Bradi5g17630		208	ND	
Bradi5g17640		211	ND	

Additional file 1. Potential CBF homologues in *Brachypodium distachyon*.

Potential CBF homologues were identified in *Brachypodium distachyon* by BLAST searches using the *Arabidopsis* CBF1, CBF2, and CBF3 proteins as baits. The *Brachypodium* proteins having amino acid sequence similarity of higher than 50% are listed. Gene names were given to 15 members of the 19 CBF homologues according to phylogenetic analysis data (see **Additional file 2**). *The cold responsiveness of each homologue has been determined by microarray assays [30]. That of the BdCBF1, BdCBF2, BdCBF3 proteins were determined by RT-PCR (this work). ND, not determined.
