# Supplementary material for: Molecular and functional characterization of cold-responsive C-repeat binding factors from Brachypodium distachyon
Source: BMC Plant Biol. 2014 Jan 9;14:15. doi: 10.1186/1471-2229-14-15 (PMC3898008; doi:10.1186/1471-2229-14-15)
Supplement: Additional file 2 — Phylogenetic analyses of Brachypodium CBF homologues. The phylogenetic relationship of the Brachypodium CBF homologues was analyzed using the MEGA5 software [32]. The Brachypodium CBF homologues were classified into 4 clades (I, II, III, and IV), among which the clade IV members are somewhat distant from the CBF homologues belonging to clades I to III. We selected one homologue that is cold-responsive from each clade and named BdCBF1, BdCBF2, and BdCBF3, as listed in bold. The remaining BdCBF proteins within each clade were serially numbered, like 2.1, 3.1, 3.2, etc. (also see Additional file 1). [file 1471-2229-14-15-S2.pdf]

Additional file 2

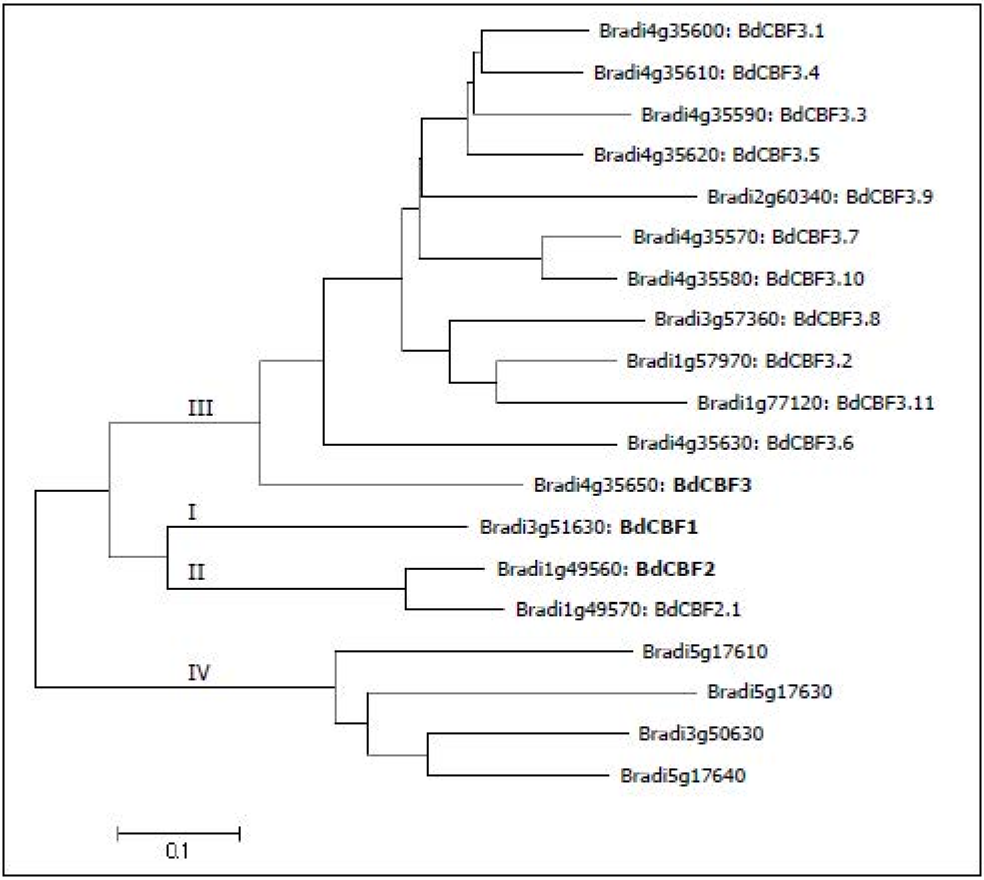

Additional file 2. Phylogenetic analyses of *Brachypodium* CBF homologues.

The phylogenetic relationship of the *Brachypodium* CBF homologues was analyzed using the MEGA5 software [55]. The *Brachypodium* CBF homologues were classified into 4 clades (I, II, III, and IV), among which the clade IV members are somewhat distant from the CBF homologues belonging to clades I to III. We selected one homologue that is cold-responsive from each clade and named BdCBF1, BdCBF2, and BdCBF3, as listed in bold. The remaining BdCBF proteins within each clade were serially numbered, like 2.1, 3.1, 3.2, etc (also see **Additional file 1**).
