# Supplementary material for: Molecular and functional characterization of cold-responsive C-repeat binding factors from Brachypodium distachyon
Source: BMC Plant Biol. 2014 Jan 9;14:15. doi: 10.1186/1471-2229-14-15 (PMC3898008; doi:10.1186/1471-2229-14-15)
Supplement: Additional file 3 — C-repeat (CRT) elements from the barley Dhn5.1 and COR14B promoters and the Arabidopsis COR15A promoter. The core CRT sequence (CCGAC), designated BS, was mutated to AAATA (mBS), as underlined, for electrophoretic mobility shift assays. [file 1471-2229-14-15-S3.pdf]

Additional file 3

Dhn5.1	BS	TTACATGCCGACACTTCCATTACATGCCGACACTTCCA
Dhn5.1	mBS	TTACATGAAATAACTTCCATTACATGAAATAACTTCCA
COR15A	BS	TTCATGGCCGACCTGCTTTTTCATGGCCGACCTGCTTT
COR15A	mBS	TTCATGGAAATACTGCTTTTTCATGGAAATACTGCTTT
COR14B	BS	ATGCTTGCCGACCTCACGTATGCTTGCCGACCTCACGT
COR14B	mBS	ATGCTTGAAATACTCACGTATGCTTGAAATACTCACGT

Additional file 3. C-repeat (CRT) elements from the barley *Dhn5.1* and *COR14B* gene promoters and the *Arabidopsis COR15A* gene promoter. The core CRT sequence (CCGAC), designated BS, was mutated to AAATA (mBS), as underlined, for electrophoretic mobility shift assays.
