# Supplementary material for: Molecular and functional characterization of cold-responsive C-repeat binding factors from Brachypodium distachyon
Source: BMC Plant Biol. 2014 Jan 9;14:15. doi: 10.1186/1471-2229-14-15 (PMC3898008; doi:10.1186/1471-2229-14-15)
Supplement: Additional file 4 — Expression of BdCBF1 gene in 35S: BdCBF1 transgenic Brachypodium plant. Transcript levels of BdCBF1 gene were determined by quantitative real-time RT-PCR (qRT-PCR) using total RNA samples extracted from 14-day-old whole plants grown on ½ X Murashige and Skoog-agar plates (hereafter referred to as MS-agar plates). Biological triplicates were averaged and statistically treated using the Student t-test (*P < 0.01). Bars indicate standard error of the mean. [file 1471-2229-14-15-S4.pdf]

Additional file 4

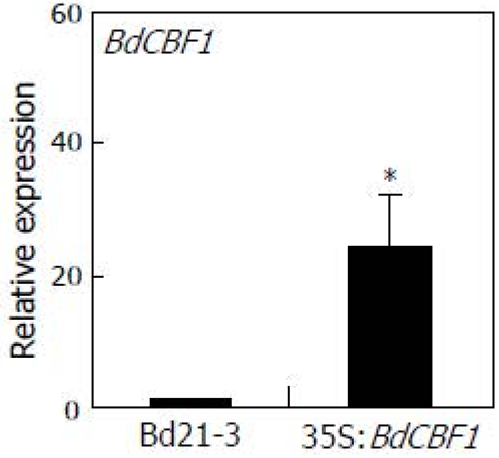

Additional file 4. Expression of *BdCBF1* gene in 35S:*BdCBF1* transgenic *Brachypodium* plant. Transcript levels of *BdCBF1* gene were determined by quantitative real-time RT-PCR (qRT-PCR) using total RNA samples extracted from 14-day-old whole plants grown on ½ X Murashige and Skoog-agar plates (hereafter referred to as MS-agar plates). Biological triplicates were averaged and statistically treated using the Student *t*-test (*P<0.01). Bars indicate standard error of the mean.
