# Supplementary material for: Molecular and functional characterization of cold-responsive C-repeat binding factors from Brachypodium distachyon
Source: BMC Plant Biol. 2014 Jan 9;14:15. doi: 10.1186/1471-2229-14-15 (PMC3898008; doi:10.1186/1471-2229-14-15)
Supplement: Additional file 5 — Expression of BdCBF1 gene in 35S: BdCBF1 transgenic Arabidopsis plants. Transcript levels of BdCBF1 gene were determined by RT-PCR using total RNA samples extracted from 10-day-old whole plants grown on MS-agar Plates. A tubulin gene (TUB) was used as RNA quality control. [file 1471-2229-14-15-S5.pdf]

Additional file 5

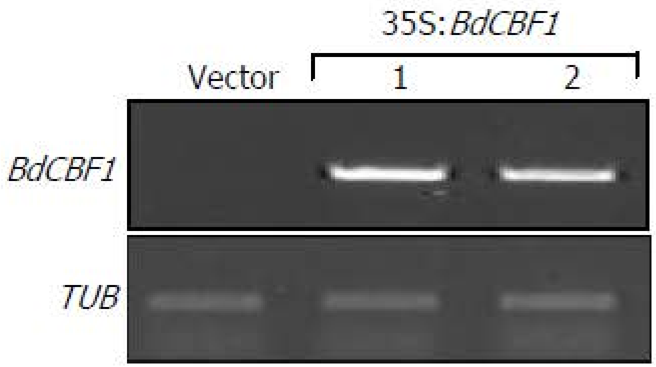

Additional file 5. Expression of *BdCBF1* gene in 35S:*BdCBF1* transgenic *Arabidopsis* plants. Transcript levels of *BdCBF1* gene were determined by RT-PCR using total RNA samples extracted from 10-day-old whole plants grown on MS-agar plates. A tubulin gene (*TUB*) was used as RNA quality control.
