# Supplementary material for: Molecular and functional characterization of cold-responsive C-repeat binding factors from Brachypodium distachyon
Source: BMC Plant Biol. 2014 Jan 9;14:15. doi: 10.1186/1471-2229-14-15 (PMC3898008; doi:10.1186/1471-2229-14-15)
Supplement: Additional file 6 — Primers used in qRT-PCR and RT-PCR. F, forward primer; R, reverse primer. [file 1471-2229-14-15-S6.pdf]

Additional file 6

Primers	Sequences	Usage
BdCBF1-F	5′-ACCCGTACTACGAGATGGGC	qRT-PCR
BdCBF1-R	5′-ATCGGAGGAGGGTCAATGAG	"
BdCBF2-F	5′-GTGGCGCAGTCGTCTTCTT	"
BdCBF2-R	5′-GCTGGTCCTGCAAGTCACAC	"
BdCBF3-F	5′-TCGTCCTCCCTCACTGACAA	"
BdCBF3-R	5′-GCGTAGTAGAGGTCCCAGCC	"
COR15A-F	5′-GCAGATGGTGAGAAAGCGAA	"
COR15A-R	5′-GGCATCCTTAGCCTCTCCTG	"
COR414-F	5′-GGGAGAGTATGGTGTATGGGCA	"
COR414-R	5′-TGATATGGCGCCACAATCA	"
KIN2-F	5′-CCAACAAGAATGCCTTCCAA	"
KIN2-R	5′-CCGATATACTCTTTCCCGCC	"
GolS3-F	5′-CAAAGTTGTCCCTCCCACAC	"
GolS3-R	5′-GAGCATGGCCAAGACAAGAT	"
BdCBF1-F	5′-CTCGCTTACCACACTCCACAC	RT-PCR
BdCBF1-R	5′-AGAGGGAAACGAAAGGGCCGGC	"

Additional file 6. Primers used in qRT-PCR and RT-PCR.
F, forward primer; R, reverse primer.
